# Determination of Community Structure and Diversity of Seed-Vectored Endophytic Fungi in *Alpinia zerumbet*

**DOI:** 10.3389/fmicb.2022.814864

**Published:** 2022-02-28

**Authors:** Kuan Yan, Zihao Pei, Lina Meng, Yu Zheng, Lian Wang, Ruizhang Feng, Quanzi Li, Yang Liu, Xianming Zhao, Qin Wei, Ahmed H. El-Sappah, Manzar Abbas

**Affiliations:** ^1^Faculty of Agriculture, Forestry and Food Engineering, Yibin University, Yibin, China; ^2^Sichuan Oil Cinnamon Engineering Technology Research Center, Yibin University, Yibin, China; ^3^State Key Laboratory of Tree Genetics and Breeding, Chinese Academy of Forestry, Beijing, China; ^4^College of Chemistry and Biological Engineering, University of Science and Technology Beijing, Beijing, China; ^5^Genetics Department, Faculty of Agriculture, Zagazig University, Zagazig, Egypt

**Keywords:** *Alpinia zerumbet* seed, endophytic fungi, high-throughput sequencing, probiotics, community structure

## Abstract

Endophytic fungi act as seed endosymbiont, thereby playing a very crucial role in the growth and development of seeds. Seed-vectored endophytic fungi establish an everlasting association with seeds and travel from generation to generation. To explore the composition and diversity of endophytic fungi in *Alpinia zerumbet* seeds, high-throughput Illumina MiSeq sequencing was employed for the following stages: fruit formation period (YSJ1), young fruit period (YSJ2), early mature period (YSJ3), middle mature period (YSJ4), and late mature period (YSJ5). A total of 906,694 sequence reads and 745 operational taxonomic units (OTUs) were obtained and further classified into 8 phyla, 30 classes, 73 orders, 163 families, 302 genera, and 449 species. The highest endophytic fungal diversity was observed at YSJ5. The genera with the highest abundance were *Cladosporium*, *Kodamaea*, *Hannaella*, *Mycothermus*, *Gibberella*, *Sarocladium*, and *Neopestalotiopsis*. Functional Guild (FUNGuild) analysis revealed that endophytic fungi were undefined saprotroph, plant pathogens, animal pathogen–endophyte–lichen parasite–plant pathogen–wood saprotroph, and soil saprotrophs. *Alternaria*, *Fusarium*, *Cladosporium*, and *Sarocladium*, which are potential probiotics and can be used as biocontrol agents, were also abundant. This study is part of the Sustainable Development Goals of United Nations Organization (UNO) to “Establish Good Health and Well-Being.”

## Introduction

Plant endophytes are endosymbionts that are integral components of a plant microecosystem. The presence of these symbiotic endophytes has been reported among all plant tissues, including roots, root hairs, stems, branches, twigs, leaves, flowers, and seeds (Gond et al., 2015; White et al., 2015). During the long-term process of coevolution, endophytes have developed a symbiotic relationship with their hosts, such as plants, to enable better colonization of land (Johnston-Monje and Raizada, 2011). Endophytic fungi are members of a large class of endophytes that play a key role in plant resistance against both biotic and abiotic stresses; promote plant growth *via* the mobilization of nutrients, phosphate solubilization, and nitrogen fixation (Rosenblueth and Martínez-Romero, 2006); and produce biologically active compounds, such as phytohormones (Compant et al., 2010; Donoso et al., 2016). The absence of these symbiotic endophytes induces serious constraints on seed germination and seedling growth (Verma et al., 2017), while their presence improves seed germination and seedling growth (Donoso et al., 2016).

Seeds are important reproductive organs of plants that fall on land, the richest source of diverse microbes. On land, seeds gather multiple endophytic microbes on their coat, which may protect them from degradation or consumption (Dalling et al., 2011; Verma et al., 2017). Seed-derived endophytes have antibacterial, antianimal feeding, and growth-promoting effects on host seedling and can effectively increase the seed germination rate, seedling survival rate, seedling root length, seedling height, and biomass accumulation (Shade et al., 2017; Berg and Raaijmakers, 2018). Endophytic symbiotic fungi establish an irreversible long-lasting association, namely, seed-vectored endophytic fungi, which travels from generation to generation (Johnston-Monje and Raizada, 2011). In Eucalyptus, rice, tea, and maize, seed-derived endophytic microbes serve as a source of future microbiota of seedlings and develop a very pleasant aroma during pil fermentation of tea (Johnston-Monje and Raizada, 2011; Yan et al., 2021a,b). Therefore, research to disclose the complex diversity of the endophytes of plant seeds, especially for medicinal and spice plants species, is critical.

*Alpinia zerumbet* (Pers.) Burtt et Smith is an important aromatic plant in China and is widely cultivated in Southern Sichuan (Tao et al., 2009). The seeds of *A. zerumbet* are a rich source of volatile oil and possess beneficial biological activities, such as antibacterial, antioxidant, analgesic, and anti-inflammatory effects, thereby providing a soothing effect to the heart and brain (Shen et al., 2010). Although *A. zerumbet* is being extensively assessed for its volatile oil contents and biological activities, very little is known about endophytic microbes. Southern Sichuan has a unique geographical location and climatic conditions. As a result, the seeds of *A. zerumbet* grown in this area may harbor comparatively different endophytes. Evaluating microbial communities *via* traditional culturing technique does not reveal the complete microbial diversity of endophytes as only 0.1–10% of microbes are culturable. The composition and diversity of fungal communities can be determined using shotgun genomic sequencing or amplicon and sequencing of internal transcribed spacer (ITS) (De Filippis et al., 2017).

Seeds of *A. zerumbet* were collected from the southern Sichuan province of China and used as the material in this study. These seeds were employed to disclose the endophytic fungal community structure and diversity at five different growth stages. We deployed a robust culture-independent method, namely, high-throughput sequencing, to evaluate the community structure, diversity, and dominant genus of endophytic fungi. Our findings are helpful for revealing the correlation between the community structure and dynamic succession of endophytes at different developmental stages of *A. zerumbet* seeds. Accordingly, these results will open new research avenues, ultimately encouraging scientists to further determine the specific role of endophytes in volatile oil production in seeds of *A. zerumbet*.

## Materials and Methods

### Experimental Materials

Seeds of *A. zerumbet* were collected at five different growth stages, including fruit formation period (YSJ1), young fruit period (YSJ2), early mature period (YSJ3), middle mature period (YSJ4), and late mature period (YSJ5) in Yibin city, Sichuan Province, China (28°53′17″N, 104°43′7″E). Seeds were packed in small polythene bags, labeled, and immediately stored at 4°C to avoid decay or loss of fungal endophytes (Chang et al., 2018). Upon arrival at the laboratory, all appendages and soil patches on the surface of seeds were thoroughly washed with running tap water (Fareed et al., 2017). Seeds of each growth period were weighed (10 g, ∼100–120 seeds), packed in sterile bags, and stored at 4°C for future use as a research material. Surface sterilization of *A. zerumbet* seeds was performed *via* soaking in 75% ethanol for 30 s and 2% NaClO solution for 2 min with gentle shaking followed by three rounds of washing with sterile ddH_2_O. To confirm the surface sterilization of seeds, 20 μl of ddH_2_O from the final rinse was streaked on three plates containing a PDA medium and incubated at 28°C in a biochemical incubator in the dark (Abbas et al., 2016; El-Sappah et al., 2021).

### DNA Extraction

To extract DNA from endophytes, 5 g of seeds was weighed on an electrical balance and immediately soaked in 25 ml of ddH_2_O in triplicate. Astragalus was divided into two halves using a sharp sterile blade, dipped in PBS solution, and placed on a magnetic stirrer for 30 min. After thorough stirring, the solution was filtered through a three-layered sterile gauze to remove coarse particles. The filtrate was subsequently centrifuged at 12,298 × *g* for 10 min at 4°C. The supernatant was discarded, and the precipitate of three replicates was mixed in one tube, which was further used for genomic DNA (gDNA) extraction using E.Z.N.A™ Fungal DNA Miniprep Kit (OMEGA, United States) according to the standard protocol (Peng et al., 2019). The quality of the extracted gDNA was confirmed by running 2 μl of each sample on 1% agarose gel and visualizing the gel under a UV light-installed gel documentation system, namely, iBright imaging system (iBright 1500, Thermo Fisher Scientific, United States) (Wang et al., 2018). The concentration of gDNA was measured using a NanoDrop 2,000 spectrophotometer at an absorption of A260 (2.1–42.5 ng/μl). The *A. zerumbet* seed sample of each stage was used for gDNA extraction in triplicate.

### Polymerase Chain Reaction Amplification and Sequencing Analysis

To amplify the ITS1 sequence of the fungal ITS region using PCR, the following primer pairs were employed: ITS1F (5′-CTTGGTCATTTAGAGGAAGTAA-3′) and ITS2R (5′-GCTGCGTTCTTCATCGATGC-3′) (Yan et al., 2021a). Each reaction comprised a 1 × polymerase chain reaction (PCR) buffer, 1 mM of dNTPs, 0.2 mM of each forward and reverse primer, 1.25 U of FastPfu DNA Polymerase (TransStart^®^, AP221-01, China), and 2 μl of DNA template, with a final volume of 25 μl achieved by adding ddH_2_O. The PCR conditions were as follows: initial denaturation at 95°C for 3 min, 32 cycles, denaturation at 95°C for 30 s, annealing at 55°C for 45 s, amplification at 68°C for 45 s, final amplification at 68°C for 20 min, and storage at 4°C for infinity (Abbas et al., 2020). To confirm amplification and amplicon size, 2 μl of each PCR product was separated on a 2% agarose gel and visualized under UV light. The PCR product was used to construct a gDNA library, which was further analyzed with robust and high-fidelity Illumina MiSeq™ PE300 sequencing.

Data for each sample were distinguished according to the sequence index and saved in fastq format. Each sample of MP or PE data had two files, fq1 and fq2, which contain the reads of both sequencing ends and the corresponding sequences. The data obtained by MiSeq sequencing were paired-end sequence data. First, according to the overlap relationship between PE reads, the paired reads were spliced (merges) into a sequence. Further, reads and their merging were quality controlled and filtered. The barcode and primer sequences were used to distinguish correct sequences and their direction to obtain optimized data. Raw reads were eliminated, duplicate reads were merged according to PE read overlap, reads quality was assessed, and splicing events were checked using fast length adjustment of short reads (FLASH-1.2.11) software. To avoid mixing samples, specific primer-based barcodes were assigned to each sample at the start and end of sequencing. Finally, we obtained enough high-quality reads; their directions were corrected and analyzed to disclose fungal diversity (Mukherjee et al., 2014).

### Sequencing and Phylogenetic Analysis

To identify OTUs, phylogenetic clustering analysis was performed using the Uparse v7.0.1090^1^ OTU clustering software tool at an identity threshold of 97% (Ye et al., 2017). The identification of chimeric sequences was performed using UCHIME v4.2 (Edgar et al., 2011) and subsequently erased from the raw data. A group of eight bases was called “word.” The total number of combinations was calculated according to the reference library and repeated 100 times. The combinations that appear 100 times were considered a bootstrap cutoff. Furthermore, the RDP classifier Bayesian algorithm was employed to measure the confidence score of the taxonomic assignment at each taxonomic level, which was 80%. Fungal community composition and scientific classifications of endophytes in *A. zerumbet* of each sample were analyzed at a standard classification system level, such as kingdom, phylum, class, order, family, genus, and species (Chen et al., 2016). The number of common and unique OTUs in each sample was counted, and a Venn diagram was constructed (Fouts et al., 2012).

### Endophytic Fungal Diversity Analysis in *Alpinia zerumbet* Seeds

Both alpha (α) and beta (β) diversities of endophytic fungi in *A. zerumbet* seeds were analyzed. The alpha diversity (α-diversity) of a microbial community involves the evaluation of sequencing depth, coverage, and comparison of abundance and diversity of seed-derived endophytes at different growth stages. We used Good’s coverage metric (C = 1-n_1_/N) to estimate sequence coverage, where n_1_ is the number of OTUs containing only one sequence and N the is total number of sequences obtained from one sample (He et al., 2020). In contrast, the beta diversity (β-diversity) involves the exploration of diversity among different species in a microbial community found at different habitats or communities. We calculated the α-diversity of three biological repeats of each sample by employing Chao 1, ACE, Shannon, and Simpson indices (Gihring et al., 2012; Rogers et al., 2016). To explore the Euclidean distances and dissimilarity index among all samples, variation in the composition of OTUs at an identity threshold of 97% was calculated in each sample by principal component analysis (PCA) and principal coordinate analysis (PCoA) (Calderón et al., 2016). To analyze the functional groups of endophytic fungi present in *A. zerumbet* seeds, all reads were analyzed in the FUNGuild database^2^ (Song et al., 2019). Comparative taxonomic analyses of the endophytic fungal community present in all samples were performed at each level of classification, and the R tool was used to construct both community structure diagrams and histograms (Lu et al., 2016; Zhou et al., 2017).

### Statistical Analysis

All data are expressed as mean values, and one-way analysis of variance (ANOVA) was used to conduct the analysis. Further, Tukey’s HSD test was performed to analyze variations among means of Shannon, Simpson, ACE, Chao, and diversity indices at a significance level of *p* < 0.05. All correlation and path coefficient analyses were performed with SPSS Statistics 20.0 software (SPSS Inc., Chicago, IL, United States) and Excel 2019.

## Results

### Sequencing Data Analysis

**FIGURE 1:**
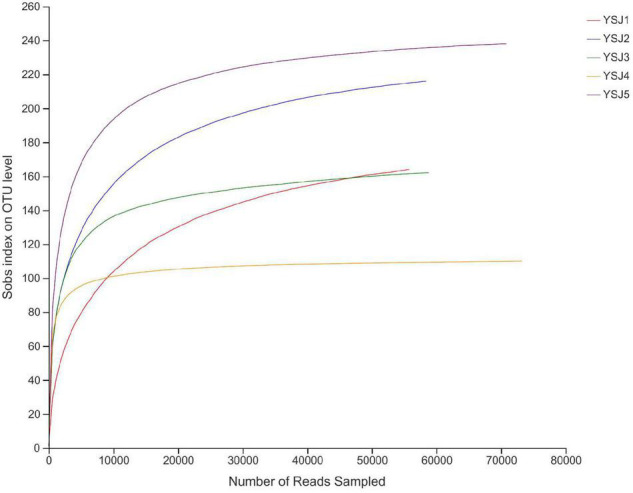
Rank abundance curves of all samples. The abscissa represents the rank number of operational taxonomic units (OTUs), and the ordinate represents the relative percentage of species at the classification level. The position of the abscissa of extension end point of the sample curve represents the number of species in each sample. Smooth curves indicate higher species diversity, while steep decline indicates a high proportion of bacterial strains and low fungal diversity.

Endophytes from *A. zerumbet* seeds were harvested, and high-fidelity MiSeq analysis was carried out after DNA extraction to discover the community structure of fungal endophytes. We obtained 171,166 reads from YSJ1, 176,695 from YSJ2, 171,928 from YSJ3, 201,842 from YSJ4, and 185,063 from YSJ5 (Table 1). From all seed samples collected at the different stages, 906,694 high-quality endophytic fungal genomic sequences were obtained with read lengths ranging from 140 to 527 bp (Table 1 and Supplementary Table 1). A group of eight bases was called “word,” and more than 60,000 combinations were obtained. The total combinations of sequences were calculated according to the “word” reference library and repeated 100 times. The number of calculations, which appeared in the same classification position for 100 times, was considered the bootstrap cutoff or confidence threshold, which was 80%. All classification results were obtained by following this bootstrap cutoff. A rarefaction curve was constructed to evaluate the sampling and sequencing depth of each sample (Figure 1). The rarefaction curves of all samples were flat and saturated, confirming that the sampling depth and sequence reads were at appropriate levels. The confidence in the endophytic fungal community structure was very high, which reflected the maximum inclusion of endophytic fungi in *A. zerumbet* seeds during sequencing.

**TABLE 1:** Average internal transcribed spacer (ITS) reads of fungal endophytes in *Alpinia zerumbet* seeds at five stages.

Sample	Reads	Total bases	Average length
YSJ1	171,166	31,768,534	183.73
YSJ2	176,695	40,193,259	227.83
YSJ3	171,928	39,236,634	228.22
YSJ4	201,842	44,929,057	221.71
YSJ5	185,063	40,008,573	213.89

### Operational Taxonomic Unit Cluster Analysis

**FIGURE 2:**
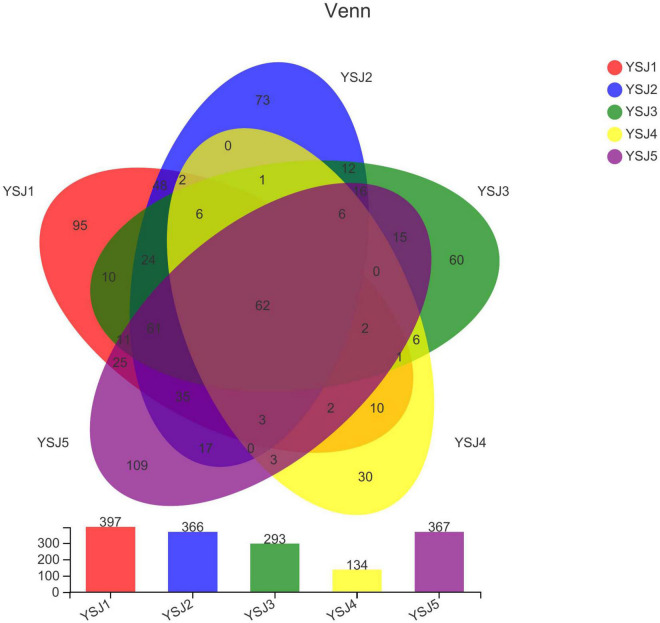
Venn diagram of OTUs. Different groups are represented by different colors, and numbers in overlapping portions represent the number of species common in all groups.

A Venn diagram was constructed to depict similar and overlapping OTUs among all *A. zerumbet* seeds samples at different classification levels (Figure 2). A total of 741 OTUs were pooled through statistical analysis at a similarity threshold of 97% and further classified into 8 phyla, 30 classes, 73 orders, 163 families, 301 genera, and 446 species. Endophytic fungal OTUs obtained from each sample of *A. zerumbet* seeds collected at different stages were 397, 366, 293, 134, and 367, respectively (Supplementary Table 1). As displayed in the Venn diagram, 241 OTUs were common between YS1 and YS2, 177 OTUs were common between YS1 and YS3, 88 OTUs were common between YS1 and YS3, and 201 OTUs were common between YS1 and YS5. The unique OTUs among all samples collected at different stages were 95, 73, 60, 30, and 109, respectively, which were 23.9, 19.9, 20.5, 22.4, and 29.7% of the total OTUs. Evidently, only 62 endophytic fungal OTUs were common among all five groups, which were 8.3% of the total number of OTUs (Figure 2). A decreasing trend of endophytic fungal OTUs at the initial stage of growth of *A. zerumbet* seeds was observed; however, a gradual increase with an increase in growth was observed, indicating a significant change in the community structure of endophytic fungi.

### Diversity Analysis of the Endophytic Fungal Community

The coverage threshold of ITS sequences obtained from *A. zerumbet* seeds was > 0.9996, indicating that it represents the true fungal populations in the microbial community of each sample (Table 2). Higher Shannon index and lower Simpson index indicate the highest diversity index of fungal community in a sample. The highest endophytic fungal community diversity was observed in samples collected at the YSJ5 growth period followed by the YSJ1 period. In contrast, the lowest fungal community diversity was observed in samples collected at the YSJ4 growth stage. Similarly, Chao and ACE indices were employed to evaluate the richness of fungal communities in all samples (Table 2). We observed the highest endophytic fungal community abundance in YSJ5 samples followed by YSJ1 samples, and the lowest fungal community abundance in YSJ4 samples. These findings indicate that the richness and diversity of the endophytic fungi of *A. zerumbet* seeds were quite different at different growth stages, with an increase followed by a decrease (Table 2).

**TABLE 2:** Endophytic community richness and diversity indices of *A. zerumbet* seeds.

Sample	Shannon	Simpson	ACE	Chao	Coverage
YSJ1	2.797 ± 0.047^a^	0.143 ± 0.035^a^	236.04 ± 8.55^a^	233.75 ± 7.44^a^	0.9996 ± 0.0001^a^
YSJ2	2.582 ± 0.042^a^	0.180 ± 0.036^a^	234.52 ± 5.27^a^	235.23 ± 4.79^a^	0.9996 ± 0.0001^a^
YSJ3	2.751 ± 0.031^a^	0.181 ± 0.033^a^	179.65 ± 10.71^a^	179.49 ± 7.13^a^	0.9998 ± 0.0001^b^
YSJ4	2.373 ± 0.070^b^	0.242 ± 0.032^b^	111.41 ± 9.54^b^	113.00 ± 3.19^b^	0.9999 ± 0.00018^c^
YSJ5	3.541 ± 0.061^a^	0.074 ± 0.010^a^	254.43 ± 12.99^a^	255.55 ± 4.67^a^	0.9998 ± 0.0001^b^

*Last column contains the average values of the diversity index. Lowercase letters indicate significant differences between samples (p < 0.05).*

### α-Diversity Analysis of Endophytic Fungal Communities

**FIGURE 3:**
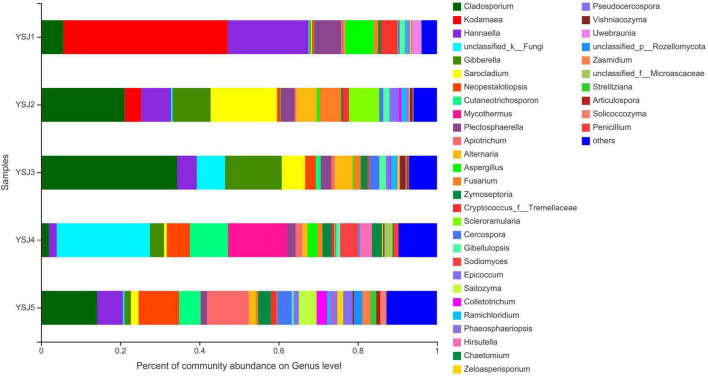
Fungal community structure bar plot analysis at the genus level. The ordinate represents sample name, and the abscissa represents the percentage of different species, which is represented by columns with different colors, sizes, and proportions of a species.

**FIGURE 4:**
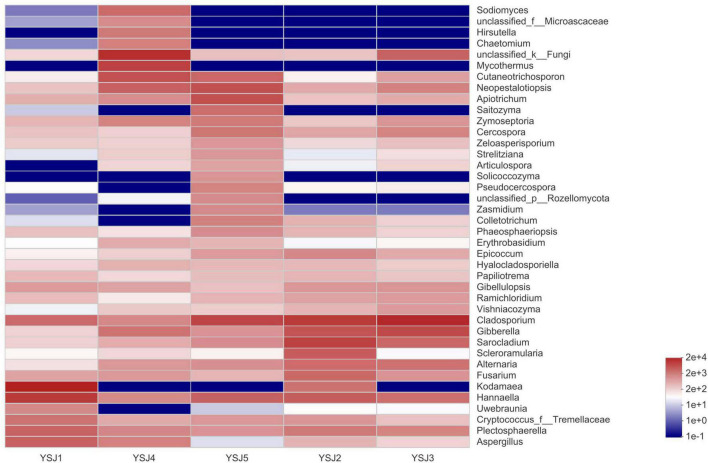
Community heatmap analysis at the genus level. The bar on the right side represents the abundance value by color gradient.

During taxonomic analysis, sequence reads were normalized at 43,847 minimum number of sequence reads per sample and a bar chart revealed 38 annotated genera of relatively high abundance and their taxonomic name, as well as relatively low abundant genera (Figure 3). Overall, the highly abundant endophytic fungal genera in all samples of *A. zerumbet* seeds were *Cladosporium* (5.64, 21.10, 34.36, 2.02, and 14.18%, respectively), *Kodamaea* (41.55, 4.22, < 0.01, < 0.01, and < 0.01%, respectively), *Hannaella* (20.36, 7.46, 4.94, 1.94, and 6.45%, respectively), *Mycothermus* (0.26, 0.43, 7.17, 23.57, and 0.45%, respectively), *Gibberella* (0.30, 9.60, 14.31, 3.51, and 1.59%, respectively), *Sarocladium* (0.31, 16.78, 5.90, 0.71, and 1.95%, respectively), and *Neopestalotiopsis* (0.43, 0.88, 2.70, 5.81, and 10.24%, respectively) (Figure 3). Evidently, the highest richness of fungal species was observed at YSJ5 followed by YSJ1. *Cladosporium*, *Hannaella*, and *Gibberella* had the highest abundance, while *Plectosphaerella*, *Vishniacozyma*, and *Ramichloridium* had the lowest abundance at each growth stage of *A. zerumbet* seeds. *Kodamaea* fungal genus had high abundance at YSJ1 and low abundance at YSJ3. Contrastingly, *Neopetalotiopsis* fungal genus had low abundance at YSJ2 and high abundance at YSJ5 (Figure 4). Thus, the growth pattern and abundance/richness of endophytic fungal genera were not the same at different growth stages of *A. zerumbet* seeds.

### β-Diversity Analysis of the Endophytic Fungal Communities

**FIGURE 5:**
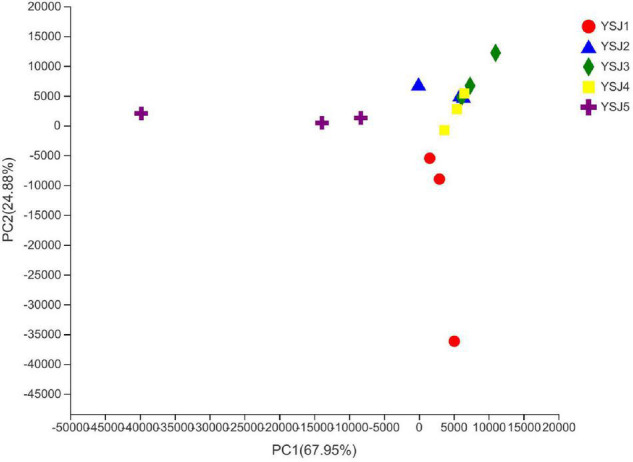
Multiple sample principal component analysis (PCA) of the OTU level. Both selected principal component axes are represented by the *x-axis* and *y-axis*, and percentage represents the difference in sample composition by principal component; scales of *x-axis* and *y-axis* represent relative distances. Samples are represented by different color points or shapes in different groups. Closeness of points or shapes represents the similarity level between fungal species composition.

**FIGURE 6:**
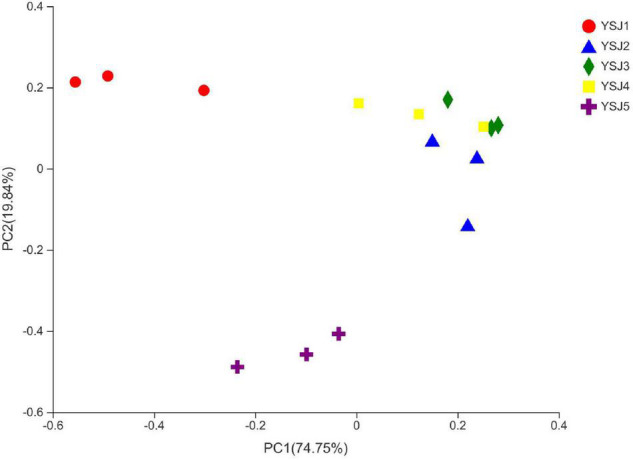
Multiple sample principal coordinate analysis (PCoA) of the OTU level. Both selected principal component axes are represented by the *x-axis* and *y-axis*, and percentage represents the difference in sample composition by principal component; scales of *x-axis* and *y-axis* represent relative distances. Samples are represented by different color points or shapes in different groups. Closeness of points or shapes represents similarity level between fungal species composition.

To explore the β-diversity, PCA (Figure 5) and PCoA (Figure 6) of samples of *A. zerumbet* seeds were performed. The highly abundant fungal genera present in all samples were mapped to construct PCA and PCoA maps, respectively. The sum of PC1 and PC2 was 92.83% in PCA and 94.59% in PCoA, indicating a high diversity in the fungal population structure in samples collected from *A. zerumbet* seeds. The samples collected at YSJ2, YSJ3, and YSJ4 were clustered closely but were far from samples collected at YSJ1 and YSJ5. Similarly, samples YSJ1 and YSJ5 were distantly clustered. These results proved the significant diversity among endophytic fungal communities in *A. zerumbet* seeds.

### Functional Guild Function Prediction of Endophytic Fungi

**FIGURE 7:**
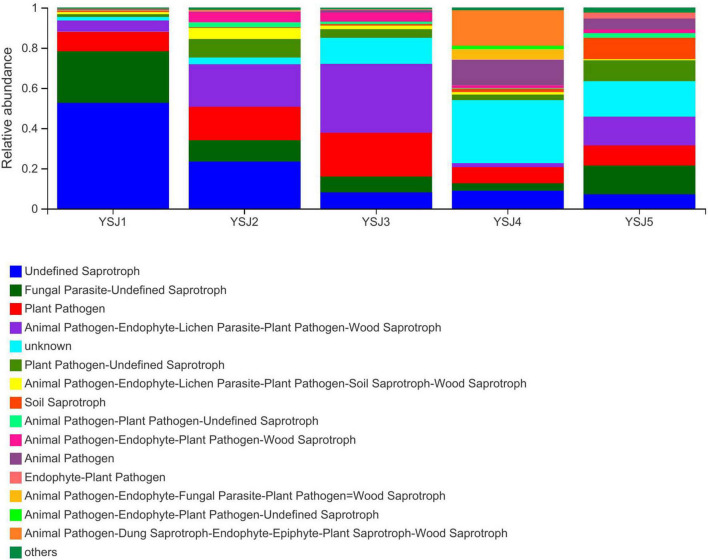
Functional Guild (FUNGuild) analysis of the fungal functional groups. Relative abundance of Guild in different groups or samples is depicted on the *x-axis* and groups or samples are presented on the *y-axis*. According to variation in the functional group, FUNGuild can calculate the abundance of each fungal species and its functional classification in each sample.

Functional Guild (FUNGuild) is a tool used for the classification and analysis of fungal communities in a microecological guild. Functional classification of fungi in a sample and abundance of fungal genera present in different samples are helpful to disclose their source and pathway (Schmidt et al., 2019). FUNGuild was used to classify fungi as undefined saprotroph, plant pathogens, animal pathogen–endophyte–lichen parasite–plant pathogen–wood saprotroph, soil saprotroph, and others (Figure 7). The source of *A. zerumbet* seeds-derived endophytic fungi differed at different growth stages; however, the environment was the richest source. The abundance of different types of fungal endophytes in samples collected from *A. zerumbet* seeds was altered at each growth stage from YSJ1 to YSJ5. Evidently, the samples collected at YSJ5 were highly abundant in endophytic fungi.

## Discussion

Normal germination of seeds of different plants is markedly dependent on the presence of diverse microbial communities (Mehra et al., 2017). Seed-derived microbial community is affected by soil, surrounding environment, host genotype, and seed quality (Klaedtke et al., 2016; Walitang et al., 2018). The diversity of microbial communities around seeds is affected by the physiological state of seed and significantly impacts seed health (Zhang et al., 2017). The presence of microbes in a plant rhizosphere has been widely assessed; however, little is known about seed-derived endophytes (Stroheker et al., 2018). Robust culture-independent Illumina high-throughput sequencing was employed to reveal the actual population, community structure, diversity, population succession dynamics, and correlation of endophytic fungi in *A. zerumbet* seeds at five different growth stages (YSJ1–YSJ5). A total of 906,694 high-fidelity fungal sequences were retrieved with an average length of 216.32 bp. A total of 745 OTUs were obtained, which were further divided into 8 phyla, 30 classes, 73 orders, 163 families, 302 genera, and 449 species. The following endophytic fungal genera, *Gibberella*, *Aspergillus*, *Alternaria*, *Fusarium*, *Cladosporium*, *Kodamaea*, and *Hannaella*, identified in *A. zerumbet* seeds were also isolated from the seeds of several species, such as *Salvia miltiorrhiza* (Barret et al., 2015; Chen et al., 2018).

Studies on different species of *Podocarpus* and *Tinospora* revealed that the geographical location and climatic conditions affect the endophytic fungal diversity in a given area (Joshee et al., 2009; Mishra et al., 2012). Additionally, metabolites stored in seeds are the richest source of nutrients and temperature for growth and reproduction of endophytic fungi (Abdallah et al., 2016). The diversity index of endophytic fungi in *A. zerumbet* seeds collected from the same area and different time periods decreased from YSJ2 to YSJ4 and then increased at YSJ5. This finding could be due to the availability of a favorable environment, such as temperature, water contents, and dry matter for fungal growth at YSJ1, which were depleted during YSJ2 to YSJ4 and stored at the final maturation stage, YSJ5 (Zhang et al., 2019). Different endophytes become dominant at different stages of seed germination due to their adaptive nature of variable internal seed environment, such as *Kodamaea* at YSJ1, *Cladosporium* at YSJ2 and YSJ3, *Mycothermus* at YSJ4, and *Cladosporium* at YSJ5; a similar trend was observed in rice seeds (Shahzad et al., 2017).

A mutual relationship exists between seeds and endophytes for their growth and the biosynthesis of secondary metabolites. In *A. zerumbet* seeds, we identified few common endophytes, such as *Alternaria*, *Fusarium*, *Pythium*, and *Cladosporium*, which were previously identified in tobacco seeds (Tang et al., 2002). The dominant genera *Alternaria*, *Gibberella*, and *Aspergillus* have certain antibacterial activities (Shan et al., 2019; Zhao et al., 2021). *Cladosporium* is a rich source of biologically active compounds (Salvatore et al., 2021). *Sarocladium* plays an efficient role in controlling soil-borne diseases (Błaszczyk et al., 2021). *Fusarium* plays a significant role in plant growth by depleting Cd^2+^, As^3+^, and Pb^2+^ contents and promoting seed germination (Jin et al., 2017). *Colletotrichum* harbors special metabolites that inhibit phosphatidylinositol 3-kinase (PI3Kα) (Yang et al., 2019). *Aspergillus* is famous for producing enzymes, such as cellulase, xylanase, manganese peroxidase (MnP), esterase (Batista-García et al., 2014), α-amylase of obligate halophilic, and lactase (Ali et al., 2015; Ma et al., 2017). These findings provide information that would be useful in further studies to determine the specific biological functions of *A. zerumbet* seed-derived endophytes.

Seeds detach from plants, disperse, and can gather pathogenic fungal microbes around them, which become active under favorable conditions, resulting in disease or decay (Song et al., 2017). Most of the endophytic fungi in *A. zerumbet* seeds were undefined saprotroph, plant pathogens, and animal pathogen–endophyte–lichen parasite–plant pathogen–wood saprotroph (Figure 7). The abundance of pathogens and saprotrophs indicates the presence of parasitized endophytes in *A. zerumbet* seeds. Noticeably, when FUNGuild was employed to explore ecological guilds to further parse OTUs of endophytic fungi in *A. zerumbet* seeds, some results were incomplete and uninterpretable. For example, *Aspergillus candidus* was only classified as a saprotroph; however, according to several reports, it is also an endophytic fungus in plants (Ma et al., 2017). Thus, the FUNGuild database needs to be updated.

## Conclusion

We explored the endophytic fungi in *A. zerumbet* seeds by deploying culture-independent, robust high-throughput sequencing of the ITS region of fungi. Although the efficacy of ITS sequencing is not 100% due to the limitations of next-generation sequencing read length, this method covers a broad range of fungal genera relative to a traditional culturing technique. *A. zerumbet* seeds were also found to harbor endophytic fungal genera that are extensively distributed in all seeds, such as *Altrnaria*, *Fusarium*, *Pythium*, and *Cladosporium*. The abundance of endophytic fungi in *A. zerumbet* seeds may have some relation with the biosynthesis of an essential oil. Many potential probiotics were identified in *A. zerumbet* seeds, including *Fusarium, Alternaria*, *Cryptococcus*, *Colletotrichum*, and *Aspergillus*, which can be further used as probiotics and biocontrol agents.

## Data Availability Statement

The datasets presented in this study can be found in online repositories. The names of the repository and accession number(s) can be found below: European Nucleotide Archive (ENA) under accession number PRJEB49388 (ERP133892).

## Author Contributions

KY, MA, AE-S, and QW conceptualized the study and wrote the original draft. KY, ZP, LM, YZ, LW, RF, QL, YL, XZ, and MA draw the figures. KY, AE-S, QW, and MA did the editing and proofreading of the study. KY and MA supervised the study. KY was the project administrator. All authors contributed to the article and approved the submitted version.

## Conflict of Interest

The authors declare that the research was conducted in the absence of any commercial or financial relationships that could be construed as a potential conflict of interest.

## Publisher’s Note

All claims expressed in this article are solely those of the authors and do not necessarily represent those of their affiliated organizations, or those of the publisher, the editors and the reviewers. Any product that may be evaluated in this article, or claim that may be made by its manufacturer, is not guaranteed or endorsed by the publisher.

## Footnotes

1
drive5.com/uparse/

2
www.funguild.org
